# Development of a diagnostic instrument for the assessment of teaching competencies in medicine (FKM_L): First results of the test statistical verification

**DOI:** 10.3205/zma001574

**Published:** 2022-11-15

**Authors:** Marianne Giesler, Maria Lammerding-Köppel, Jan Griewatz

**Affiliations:** 1 Freiburg, Germany; 2 Tübingen, Germany; 3 Kompetenzzentrum für Hochschuldidaktik in Medizin Baden-Württemberg, Tübingen, Germany

**Keywords:** teaching competencies in medicine, self-assessment, self-reflection, diagnostic of competencies

## Abstract

**Introduction:**

This project report describes the development of the *Questionnaire for the Assessment of Teaching Competencies in Medicine* (FKM_L) and the analysis of some of its psychometric properties. The design of the FKM_L is based on the model of* Core Competency for Teachers in Medicine* (KLM) model of the *GMA Committee on Personnel and Organizational Development in Teaching*.

**Methods:**

Global questions and in-depth items were formulated for each of the sub-competencies of the six core competencies of the KLM model. Depending on the number of sub-competencies, there are 3-4 subscales for each core competency, comprising 69 items in total. Data from 90 participants of medical didactic courses were analysed. Item analyses supported the hypothesized scales.

**Results:**

The internal consistencies (Cronbach's alpha: CR-α) of the 22 subscales ranged from CR-α=.70 to CR-α=.93, and the item difficulty indices of the subscales ranged from 18% to 89%. For 2 subscales, some items had a difficulty index of more than 80%, and for 3 subscales, the difficulty index of some items was less than 25%.

**Conclusions:**

The FKM_L was developed to assess individual and group profiles of teachers’ competence in medicine. The results of this first psychometric analysis are promising: With the help of the FKM_L, teachers can learn about and reflect on aspects of their teaching competencies in the context of medical didactic courses. Based on their FKM_L profiles, they can decide whether they want to selectively optimize their competence characteristics. For providers, the FKM_L is suitable as a screening tool to identify, among other things, gaps in the training offered. Further analyses are necessary to check limitations identified in some scales and to improve individual items. In addition, research on the construct and criterion-related validity of the instrument is required.

## Zusammenfassung

**Einführung:**

In diesem Projektbericht wird die Entwicklung und teststatistische Überprüfung des *Fragebogens zur Erfassung von Lehrkompetenzen in der Medizin* (FKM_L) beschrieben. Die Basis für die Konzeption des FKM_L bildete das Modell *Kernkompetenzen für Lehrende in der Medizin* (KLM) des *GMA Ausschusses Personal-/Organisationsentwicklung in der Lehre*.

**Methodik:**

Für die Teilkompetenzen der sechs Kernkompetenzen des KLM-Modells wurden jeweils Globalfragen und vertiefende Items formuliert. Je Kernkompetenz gibt es in Abhängigkeit von der Zahl der Teilkompetenzen 3-4 Subskalen, die 69 Items umfassen. Alle Skalen wurden itemanalytisch überprüft. Analysiert wurden Daten von 90 Teilnehmerinnen und Teilnehmern medizindidaktischer Kurse.

**Ergebnisse:**

Die inneren Konsistenzen (Cronbachs Alpha: CR-α) der 22 Subskalen liegen zwischen CR-α=.70 und CR-α=.93, die Schwierigkeitsindices der Items der Subskalen zwischen 18% und 89%. Bei 2 Subskalen wiesen einige Items einen Schwierigkeitsindex von über 80% auf, bei 3 Subskalen betrug der Schwierigkeitsindex einiger Items weniger als 25%.

**Schlussfolgerung:**

Der FKM_L wurde zur Erfassung von individuellen und gruppenbezogenen Kompetenzprofilen von Lehrenden in der Medizin entwickelt. Die Ergebnisse dieser ersten teststatistischen Analyse sind vielversprechend: Mit Hilfe des FKM_L können Lehrende im Rahmen medizindidaktischer Kurse Aspekte ihrer Lehrkompetenzen kennenlernen und reflektieren. Anhand ihrer FKM_L-Profile können sie entscheiden, ob sie ihre Kompetenzausprägungen punktuell optimieren möchten. Für Anbieter eignet sich der FKM_L als Screening-Instrument, um u.a. Lücken im Ausbildungsangebot festzustellen. Weitere Analysen sind notwendig, um festgestellte Einschränkungen bei einigen Skalen zu überprüfen und einzelne Items anzupassen. Darüber hinaus ist eine Überprüfung der Konstrukt- und der kriterienbezogenen Validität des Instruments erforderlich.

## Introduction

### General background

Studies show that a teacher's subject-matter expertise alone is not a sufficient condition for the learning success of individuals being taught [1]. Other subject-independent characteristics and competencies can significantly influence learning development [2]. For school and university settings, conceptual approaches are available that describe competencies teachers should possess in order to cope well with the many and multifaceted tasks and challenges of teaching [3]. For the field of medicine, the* GMA Committee on Personnel and Organizational Development in Teaching* derived a model based on the *Six Core Teaching Competencies for Medical Educators* by Srinivasan et al. [4] that describes *core competencies for teachers in medicine* (KLM) [2]. This model provides a framework of orientation for medical didactic competencies and indicates what a teacher’s qualification profile might look like. For providers of medical didactic qualification programs, the KLM model can be very helpful in orienting their continuing education and training programs in terms of content.

However, the KLM model itself cannot yet be used to determine the extent to which individual teachers possess the competencies described and/or to which extent the medical didactic course offerings address these competencies. The core competencies are not described in a sufficiently differentiated manner for this purpose. However, they provide a good basis for developing an appropriate diagnostic tool.

#### Diagnosis of the degree of competence

For the diagnosis of the degree of competence or for determining the individual teacher's ability to act in complex (authentic) situations, external and self-assessments can be considered, which can be carried out formatively as well as summatively by means of observations, e.g., by checklists, or by using psychometric procedures [5], [6], [7], [8], [9], [10], [11]. Both methods are subject to random and systematic errors, i.e., the measurement results cannot be perfectly reliable and valid [8]. Self-assessments in particular have a reputation for limited reliability and, accordingly, limited validity [12], [13]: In most cases, evidence is found that those who prove to be least competent are also those who are less accurate in assessing themselves [14], [15]. There is also evidence that highly competent individuals may tend to underestimate their performance [16]. Studies also show that women tend to underestimate their performance [17]. In addition, women's and men’s self-assessments may differ depending on the domain of competence [18]. Finally, there is also evidence that practical skills are better assessed than knowledge-based activities [14]. However, self-assessments can be improved by feedback [14], [19]. This can be achieved by providing feedback on the standards to be achieved and offering opportunities for comparison purposes (individual and social) [12], [14], [20]. If the goal is to determine what is required to provide individually suitable offers for further qualification, the aggregation of self-assessment data may allow reliable diagnoses [13].

#### Objective

Up to now, there has been no suitable instrument available for the assessment of teaching competencies in medical university teaching that would identify individual and general approaches for the further development of competencies. Therefore, based on the KLM model described above, a diagnostic instrument for the *Assessment of Teaching Competencies in Medicine* (FKM_L) was developed. This project report describes the development of this instrument and presents first results on its psychometric properties.

## Methods

### Development of the questionnaire

The *Core Competency Model for Teachers in Medicine* (KLM) served as the basis for our development of the *Questionnaire for the Assessment of Teaching Competencies in Medicine* (FKM_L). In this model, sub-competencies are derived from six fields of competence, for each of which learning objectives and examples of application are also described. The FKM_L is structured similarly. In a first step, so-called global items were formulated for the individual sub-competencies of the six competence fields. In a second step, in-depth items were developed for these global items, taking into account the respective learning objectives and examples of application as well as the expertise and experience of the questionnaire developers (see table 1 (Tab. 1) and table 2 (Tab. 2)), which were then combined into subscales. In formulating the items, attention was paid to conclusiveness and comprehensibility [21]. In addition, participants were asked for feedback on the usefulness of their use of the FKM_L immediately after having responding to it. This information was included in the further development of the instrument.

The FKM_L includes 22 global items and 69 in-depth items, each of which is to be responded to on 5-point Likert-type scales (“1” not given at all to “5” given to a very high degree). Only the end points of the scales are semantically anchored.

For the development of the first version of this questionnaire, several meetings of the authors were held in the period from 2018 to 2019. Further revisions were made by way of circulation. Since the middle of 2019, the FKM_L has been tested in the context of medical didactic offers of the *Competence Center for University Teaching in Medicine Baden-Württemberg in Tübingen*.

#### Sample

The analyses were based on the data of two groups of participants of medical didactic courses of the *Competence Center for University Teaching in Medicine Baden-Württemberg*. These competence-oriented courses [22] were held at different faculties in Baden-Württemberg (Freiburg, Mannheim, Tübingen) and included participants mainly from faculties in Baden-Württemberg. In the winter semester 2019/2020, 29 participants of the courses *Medical Teaching Qualification I* (MQ I, basic qualification) and 12 of the courses *Medical Teaching Qualification II* (MQ II, advanced qualification) completed the FKM_L voluntarily. In the winter semester 2020/2021 and summer semester, there were 49 participants who completed the FKM_L as part of taking the *Medical Teaching Qualification I* courses.

Because of the small course sizes, we initially refrained from systematically collecting information on age and gender for data protection reasons. In subsequent surveys, this information was obtained on a voluntary basis along with consent forms. Therefore, more detailed information on gender and age is available from only 50 participants. At the time of the survey, these participants were on average 37 years old (SD=5.95, Md=35, Mo=33), 41% are women.

The FKM_L is used immediately at the beginning of the courses (especially MQ I) as a didactic tool with the aim of introducing the topic of competencies and reflecting on the expressions of one's own teaching competencies. At later points in the course or in the subsequent advanced level, further assessments are carried out. To be able to record individual developments over time, reproducible anonymous codes can be used for the respondents.

#### Statistical analyses

Within the framework of item analyses according to the concept of so-called *Classical Test Theory*, corrected item total correlations as measures of discriminatory power and item difficulty each were calculated for every item. In addition, Cronbach's α (CR-α) was calculated for each competence scale as a measure of internal consistency.

The concept of item difficulty has originally been introduced in the context of achievement test construction. In this context, item difficulty is estimated using the so-called *difficulty index*. This is defined as the percentage of test takers who solve a test item [23]. It should be noted that a numerically high value of the difficulty index indicates that the task or item in question is “easy” because a high percentage of respondents were able to solve it. Correspondingly, a numerically low difficulty index indicates the presence of a “difficult” item, insofar as it could only be solved by a low percentage of those tested. In order to determine the difficulty of items from self-assessment questionnaires with multiple graded response options, as in the present case, the following formula can be used to calculate the difficulty index, following Döring and Bortz [24]: (M_i_ - 1 / k - 1) * 100, where M_i_ denotes the mean of the item in question and k the number of – coded continuously starting with 1 – levels of the response scale. 

As measures of discriminatory power, item total correlations indicate how highly an item correlates with the total score of the associated scale or how well an item measures the construct to be captured by the scale [24], respectively. Corrected item total correlations <.30 are considered low [25]. Values between .30 and .50 are considered medium, and values greater than .50 are considered high.

There is a paraboloid relationship between discriminative power and difficulty [23], i.e., if the difficulty of an item is low, the discriminative power is also low, at an intermediate difficulty of 50% the discriminative power reaches its maximum, thereafter, as the difficulty increases, the discriminative power decreases again. According to Lienert [23], however, medium difficulty does not necessarily mean good discriminatory power.

The following limits for CR-α are generally used to describe reliability levels: high reliability >.90 and low reliability <.80. However, the meaning of these values may depend on the context and these limits should therefore not be applied too rigidly [24], [25].

Statistical analyses were performed using the statistical program SPSS, version 26.

## Results

### Item analyses

The scale scores determined for the individuals represent sum values that were divided by the number of items in a scale. Accordingly, both the scale scores of the individuals and the means of the scales calculated for the groups can vary between 1 and 5. A high scale score thus means a high level of self-assessed competence in relation to the respective competence field (see table 3 (Tab. 3)).

As shown in table 3 (Tab. 3), all 22 subscales formed from the in-depth items show internal consistencies above CR-α=.70. Since it is generally recommended to form scales with at least three items, one item was added to the subscale MH04 of the competence domain *Didactical Activities in Medicine* from WS 2020/2021. This led to an increase in the discriminatory power of the items and the reliability of this subscale.

Corrected part-whole correlations between items and their respective scale and difficulty indices were calculated for the items of all subscales. In addition, the mean of the difficulty indices was determined for each subscale. All corrected part-whole correlations are >.30. Concerning the difficulty indices, the results show that for two competency domains one subscale each (LO01, PH02) has a mean difficulty index of >80%, i.e. the respective subscales contain rather easy items. In two other competence areas there are also subscales including items with difficulty indices of up to 25% (SL02, RW03), i.e. they are to be regarded as rather difficult.

Further results, which are not presented in detail here, show that the global items, with two exceptions, always correlate most highly and significantly with the subscales assigned to these items. However, similarly high significant correlations are also found with subscales of other competency domains. The correlations of the global items with the associated subscales shown in table 3 (Tab. 3) range from r=.32 to r=.80.

A comparison of the mean scores for the global and subscales presented in table 3 (Tab. 3) shows that the competence domains differ with regard to the level of competence respectively.

#### Intercorrelations of the overall scales

The overall scales of the FKM_L correlate moderately to highly with each other (see table 4 (Tab. 4)). The highest correlations exist between the overall scale *Social and Communicative Competence* (KK) and the overall scale *Student Centred Learning* (LO) as well as between the overall scale *Reflection and Further Development of Own Teaching Practice* (RW) and the overall *System-based Teaching and Learning* (SL) scale.

#### Examples of evaluations 

**Figure 1:**
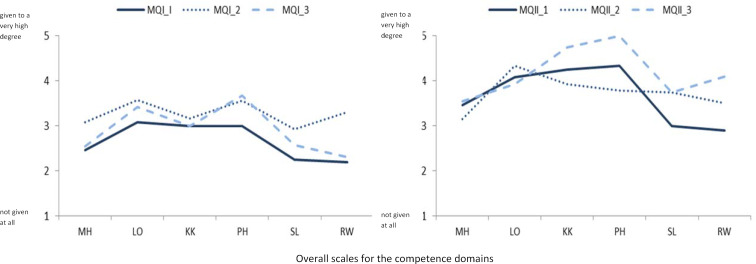
Scale values for the overall scales of the six competence domains for three different participants in each of the courses MQ I and MQ II

To illustrate, figure 1 (Fig. 1) shows scale scores of the total scales of the six competency domains for each of three randomly selected participants of the MQ I and MQ II courses. Differences between the scale scores can be seen both within and between the competence fields. With one exception, the scale scores of the participants in the MQ II course are all above the scale scores of the participants in the MQ I courses. Accordingly, an analysis of the group means reveals significant differences between the courses for four of the six overall scales.

**Figure 2:**
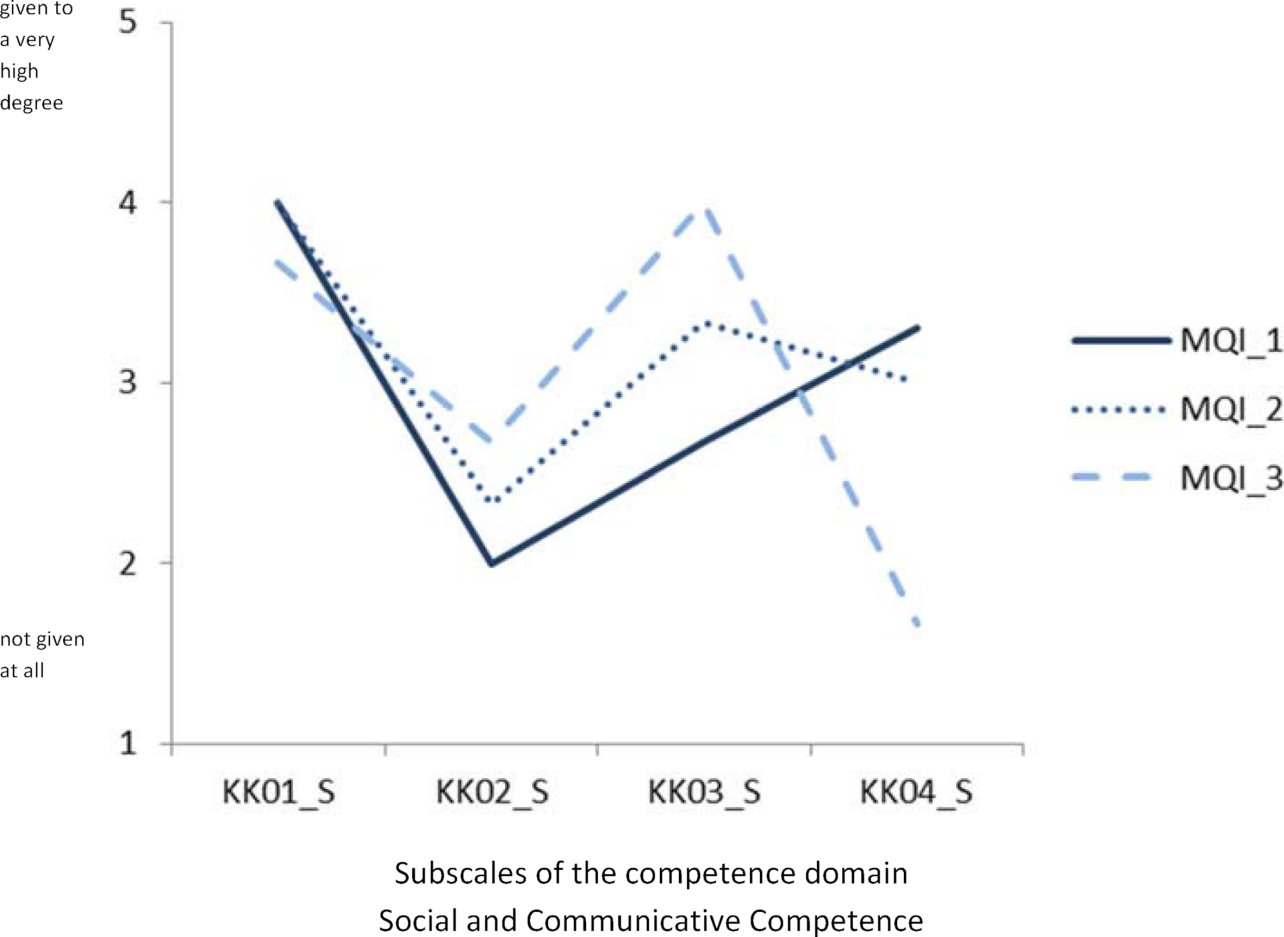
Scale values for the subscales of the competence domain Social and Communicative Competence for three participants of the MQ I courses

The illustration of the subscale scores for the three randomly selected participants of the MQ I courses for the competence domain* Social and Communicative Competence* (see figure 2 (Fig. 2)) shows intraindividual and interindividual competence level differences within this competence domain. In the context of counselling events, it can be useful to look at differences of an individual within a subskill at the single-item level. This can be demonstrated by the example of subscale KK04 on constructive feedback for person MQ I_3, who ticked the three items of this scale as follows:

“1” You follow common feedback rules when giving feedback.

“3” You recognize and use appropriate times for feedback discussions.

“1” You know and use various feedback tools.

These statements may indicate that the person already holds feedback discussions in his or her (teaching) practice, but so far has no knowledge of feedback rules and feedback instruments. In a post-course survey, one would expect these items to be rated more strongly as true. However, a reliable interpretation of such differences requires the determination of appropriate confidence intervals.

## Discussion

The analyses of psychometric properties of the FKM_L yielded mostly satisfactory results. In the following, the results are discussed separately for the subscales, overall scales, and global items as well as for the examples of evaluations. 

### Subscales

For each of the six competence areas, three to four subscales were formed to capture sub- competencies. Since internal consistencies between CR-α=.60 and CR-α=.70 are considered sufficient for group comparisons [23], [26] and reliabilities of CR-α=.80 are considered good [27], the coefficients (CR-α=.71 to CR-α=.93) obtained for the 22 subscales are in an acceptable to good range.

The discriminatory power of an item shows how well the response to the item predicts the corresponding scale sum value [24]. All the discriminatory power values determined for the subscales, with values between .34 and .91, are located in a consistently acceptable to satisfactory range. To determine whether a differentiation between persons with different levels of competence is possible with the individual items, difficulty indices were calculated for the items of the subscales. In addition, for each subscale the mean of the difficulty indices of their respective items was determined. According to Döring and Bortz [24], extremely difficult items (difficulty index <20%) as well as extremely easy items (difficulty index >80%) are to be regarded as providing little information on their ability to differentiate between respondents. The results show that the latter applies to one subscale each of the competence fields *Student Centred Learning and Role Model and Professional Behaviour*. A closer look at the items suggests that this is due to the fact that the items are phrased in a comparatively global way (e.g. “They value mutual respect”) and may possibly encourage self-serving answers. In three of the subscales, items have difficulty indices between 18% and 25%, i.e. only a maximum of 25 percent of the participants have ticked the items as applicable. Two of these subscales are assigned to the competence field of *System-based Teaching and Learning*, one subscale to the competence field of *Reflection and Further Development of own Teaching Practice*. The relevant items of these subscales usually address active participation in the planning and implementation of the curriculum. However, the participants of the medical didactics courses are heterogeneous in many ways. The participants come from different subjects, are active in different positions and functions and have been able to gain experience in teaching for more or less time. It is thus understandable that they can assess the items described above as applicable to varying degrees depending on their background. The subscales with too easy or too difficult items can nevertheless be used as a didactic tool to reflect on the respective teaching competences. Nevertheless, further analysis should be conducted to possibly supplement and/or reformulate the items of these subscales. Additional cognitive pre-tests might provide valuable clues as to how these items could be reworded.

#### Overall scales (core competencies)

For each competence domain of the FKM_L, the items of the subscales were combined into overall scales. These overall scales show consistently good internal consistencies, which is understandable since an extension of scales with additional items usually leads to higher reliability [23]. 

The FKM_L was developed based on the model of *Core Competencies for Teachers in Medicine*. Already during the development of this model, it was found that some sub-competencies and learning objectives could be assigned to different competence fields [2]. Therefore, the moderate to high intercorrelations of the total scales can be explained well (see table 4 (Tab. 4)). As determined from the correlations between the total scales, the proportions of their common variance (r^2^) ranged between .10 and .64. 13 of 15 of these proportions were <.50, so that it can be assumed that the overall scales capture different facets of teaching competence. However, the very high intercorrelation between the overall scales *Reflection and Further Development of own Teaching Practice* (RW) and *System-based Teaching and Learning* (SL) suggests considering to either combine or shorten these two scales.

#### Global items

Most global items correlate significantly, as expected, although sometimes not substantially high (r>.50) with their associated subscales (see table 3 (Tab. 3)). In addition, global items of a respective competence domain often also correlate significantly and at comparable levels with the subscales of the other competence domains. This suggests that the global items have low discriminant validity, so that diagnostics on the level of the global items is currently not recommended. For this reason, but also in order to shorten response time and to avoid negative effects possibly associated with response load, these items were dispensed with from WS 2020/2021 onward.

#### Examples of evaluations 

With the examples of individual evaluations given, inter- and intra-individual differences in the competency characteristics are apparent. As a next step, comparison values of appropriate reference groups will be provided. However, this requires a broader data base.

#### Feedback on the questionnaire

The feedback of the participants of the different courses referred predominantly to the length of the questionnaire, i.e., to the number of items and the length of the wording of individual items. It was suggested on various occasions that the completion time should be increased to allow a more thorough assessment of one’s competencies. In addition, the use of terms not understandable without further prior information was criticized. The manner of presentation (especially the assignment of items to competencies vs. arbitrary arrangement; size of screen pages) was also addressed. Short additional information in the form of marginal notes on the reading of certain items was desired in order to make the assessments more reliable.

#### Validity

So far, steps have only been taken to ensure the content validity of the FKM_L by taking the KLM model as the basis for development. Furthermore, the authors wrote and assigned the items by drawing on theoretical arguments [24], [25]. However, a thorough examination of the construct and criterion validity of the instrument is still pending.

## Conclusion

In summary, based on the presented results, the FKM_L can already be used for individual and group diagnostics despite slight limitations: Teachers can use their FKM_L profiles to decide whether they want to review or optimize their competency characteristics in one respect or another, and faculty developers can use the FKM_L as a screening tool to identify participants' needs and gaps in their qualification offerings. In addition, the assessment of competencies and their results can be used as a didactic tool in medical didactic courses, e.g., to raise awareness and reflect on individual teaching competencies and their development needs.

## Funding

The study was conducted within the context of the BMBF-funded joint project MER*LIN* (Medical Education Research –* Lehrforschung im Netz BW*) of the medical faculties of Freiburg, Heidelberg, Mannheim, Ulm and Tübingen under the leadership of the Freiburg and Tübingen sites. Grant number TÜ: 01PL17011A, FR: 01PL17011B

## Competing interests

The authors declare that they have no competing interests. 

## Figures and Tables

**Table 1:**
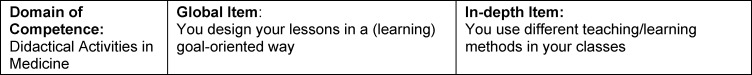
Examples of competence domain, global item and in-depth item

**Table 2:**
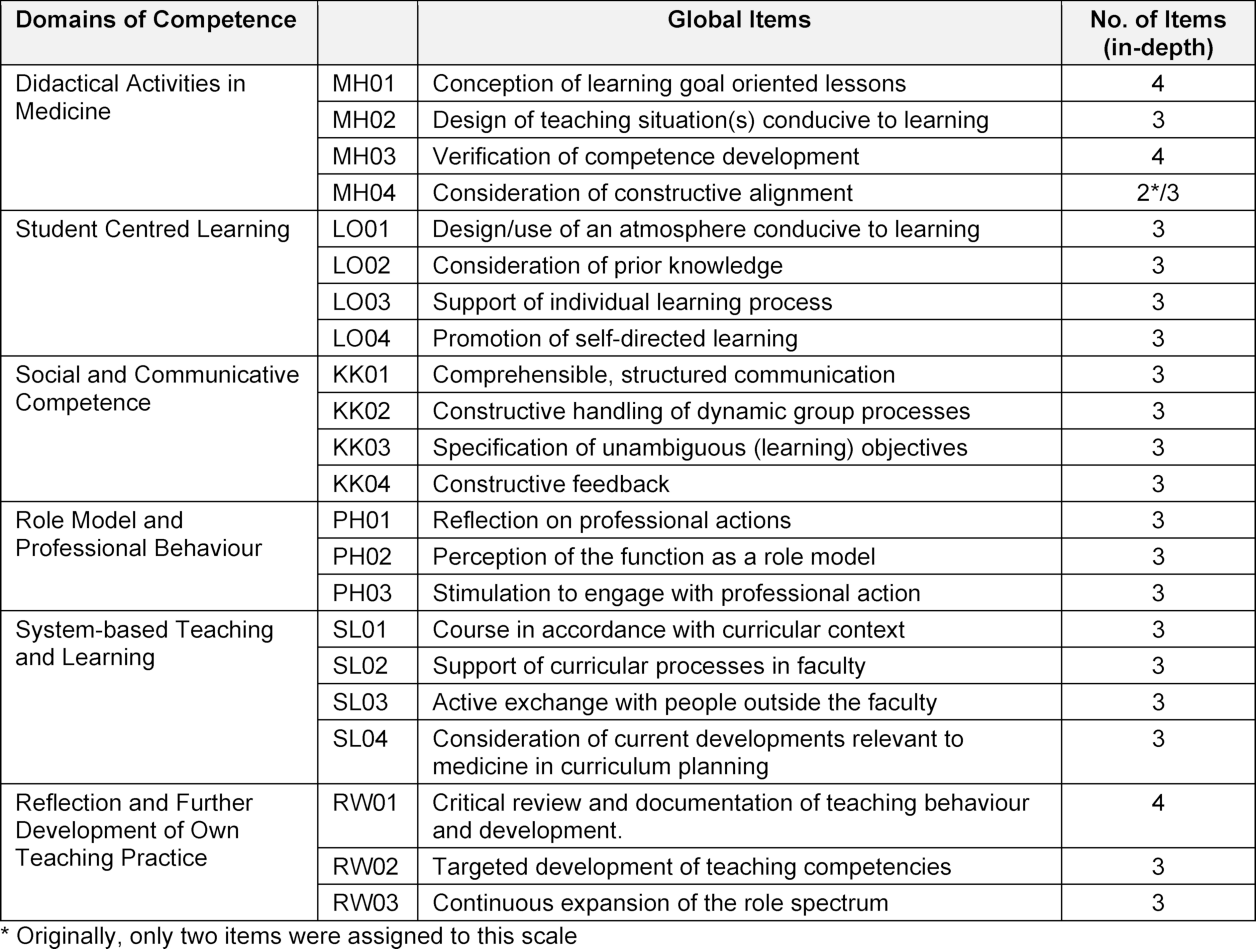
Design and structure of the FKM_L

**Table 3:**
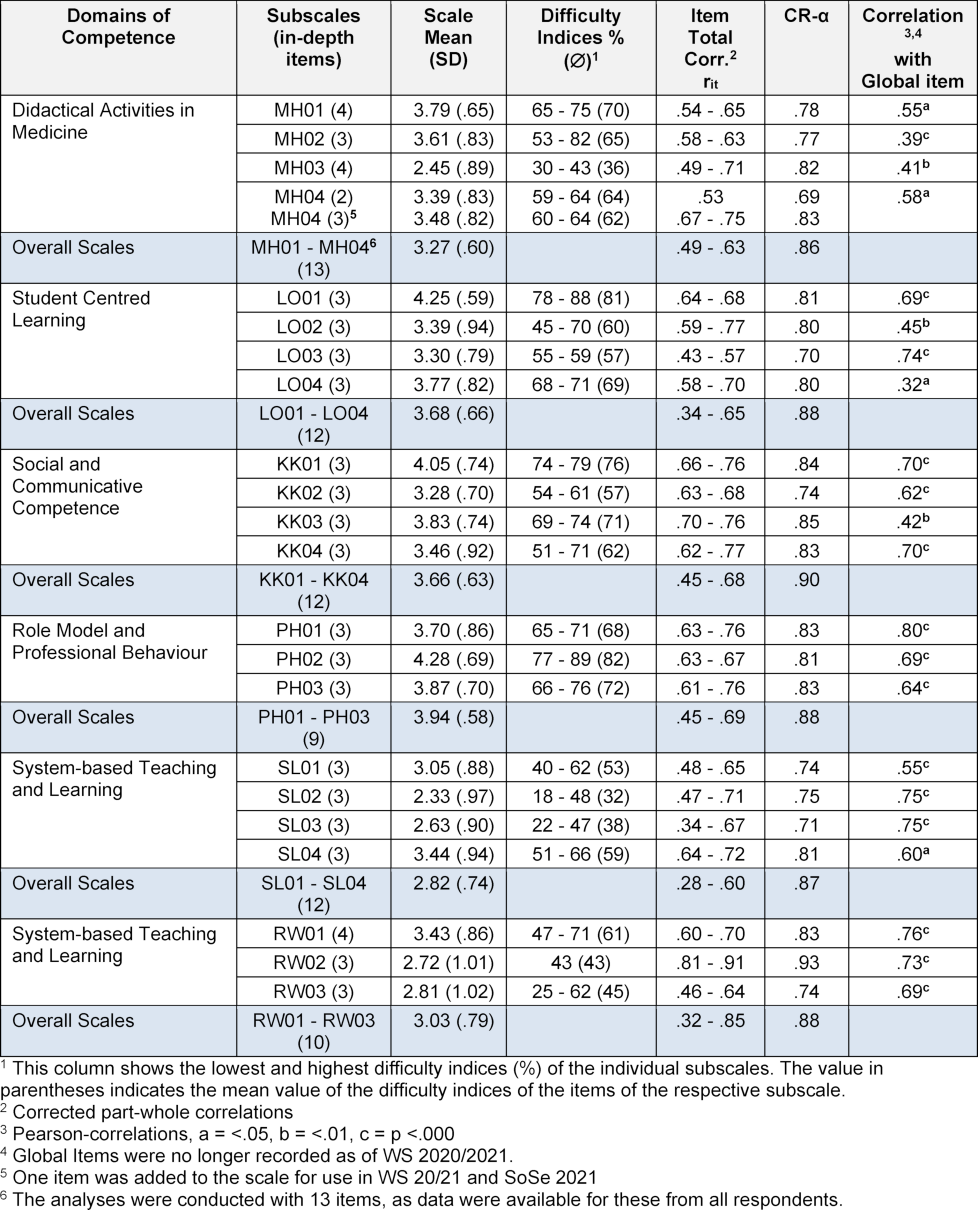
Results of the item analyses for the subscales formed with the in-depth items and for the overall scales for each domain of competence, and correlations between each subscale and the global items of a respective competence domain (N=90)

**Table 4:**
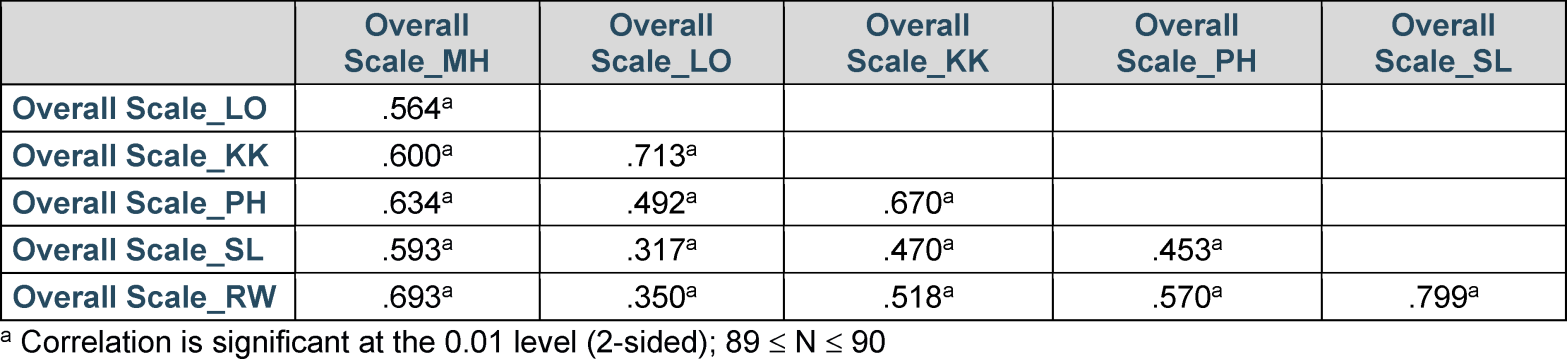
Intercorrelation of the Overall Scales of the FKM_L
